# Association between vitamin D deficiency and clinical outcome in patients with COVID-19 in the post-Omicron phase

**DOI:** 10.3389/fnut.2025.1583276

**Published:** 2025-06-18

**Authors:** I-Wen Chen, Ting-Sian Yu, Yi-Chen Lai, Chih-Ping Yang, Chia-Hung Yu, Kuo-Chuan Hung

**Affiliations:** ^1^Department of Anesthesiology, Chi Mei Medical Center, Liouying, Tainan, Taiwan; ^2^School of Medicine, College of Medicine, National Sun Yat-sen University, Kaohsiung, Taiwan; ^3^Department of Anesthesiology, E-Da Hospital, I-Shou University, Kaohsiung, Taiwan; ^4^Department of Anesthesiology, Chi Mei Medical Center, Tainan, Taiwan

**Keywords:** COVID-19, mortality, pneumonia, post-Omicron phase, vitamin D deficiency

## Abstract

**Background:**

Vitamin D deficiency (VDD) has been associated with adverse outcomes in COVID-19 patients during the early pandemic phases, but whether this association persists in the post-Omicron era remains uncertain. This study aimed to investigate the evolving relationship between VDD and COVID-19 outcomes across pandemic phases using a large healthcare database.

**Methods:**

We conducted a retrospective cohort study using the TriNetX Analytics Network, analyzing propensity-matched cohorts comprising 24,236 pairs from the post-Omicron phase (June 2022–December 2023) and 22,638 pairs from the pre-Omicron phase (January 2020–December 2021). VDD was defined as a serum 25-hydroxyvitamin D level < 20 ng/ml, with vitamin D-sufficient patients (≥30 ng/ml) serving as controls. The primary outcome was 30-day all-cause mortality, with secondary outcomes including acute kidney injury, respiratory failure, pneumonia, sepsis, and ICU admission.

**Results:**

The 30-day mortality in VDD vs. vitamin D-sufficient patients decreased from 1.43% vs. 0.39% [odds ratio (OR), 3.67; 95% CI, 2.90–4.64; *p* < 0.001] in the pre-Omicron phase to 0.89% vs. 0.49% (OR, 1.82; 95% CI, 1.46–2.28; *p* < 0.001) in the post-Omicron phase. Similar risk attenuation was observed across all secondary outcomes, including acute kidney injury (OR, 2.11; 95% CI, 1.92–2.31 vs. OR, 1.41; 95% CI, 1.29–1.54; both *p* < 0.001), respiratory failure (OR, 1.66; 95% CI, 1.44–1.92 vs. OR, 1.34; 95% CI, 1.16–1.54; both *p* < 0.001), and pneumonia (OR, 1.34; 95% CI, 1.16–1.55 vs. OR, 1.23; 95% CI, 1.07–1.42; *p* < 0.001 and *p* = 0.004, respectively). Risk factor analysis identified several significant mortality predictors among patients with VDD in the post-Omicron phase, including malnutrition (OR, 4.34; 95% CI, 3.18–5.92; *p* < 0.001), liver disease (OR, 3.08; 95% CI, 2.23–4.25; *p* < 0.001), and neoplasms (OR, 2.63; 95% CI, 2.01–3.45; *p* < 0.001).

**Conclusion:**

VDD continues to be associated with adverse COVID-19 outcomes in the post-Omicron phase, albeit with a reduced magnitude. These findings support the importance of vitamin D screening in high-risk COVID-19 patients, while emphasizing the need for adaptive risk assessment strategies that incorporate both established and emerging risk factors in the current pandemic landscape.

## 1 Introduction

Since its emergence, the COVID-19 pandemic has undergone significant changes, with the Omicron variant and its subvariants becoming the dominant global strains by 2022 (1, 2). Although vaccination campaigns and natural immunity have altered the impact of the disease, identifying modifiable risk factors remains crucial for improving patient outcomes (3, 4). Previous studies during the early pandemic phases have suggested that vitamin D deficiency (VDD) is associated with increased COVID-19 severity and mortality (5–8). The biological plausibility of this association is supported by the diverse roles of vitamin D, including regulation of immune function, suppression of inflammatory cytokines, and preservation of pulmonary barrier integrity (9, 10). This association has been further substantiated in recent studies. For instance, Renieris et al. (11) demonstrated that VDD is associated with worse outcomes in COVID-19 patients and showed that vitamin D supplementation reduced lung inflammation and cytokine expression in a COVID-like animal model, possibly through the attenuation of tissue-specific hyperinflammation. Khalil et al. (12) demonstrated that vitamin D downregulates the NLRP3 inflammasome pathway in patients with severe COVID-19, resulting in reduced expression of IL-1β and other key inflammatory mediators, highlighting its anti-inflammatory effects.

The emergence of Omicron variants, characterized by distinct transmission patterns and clinical presentations, has fundamentally altered the COVID-19 landscape (13, 14). For example, studies early in the pandemic showed that preoperative COVID-19 infection greatly increased postoperative risks (15–18), leading to strict screening and waiting periods. However, in the Omicron era, recent studies (19–21) have suggested that these risks have declined, with infected patients now experiencing outcomes comparable to those without infection, thereby challenging the earlier clinical guidelines. However, a recent study by Chen et al. (22) reported that pre-infection vitamin D status was significantly associated with the incidence, severity, and recurrence of Omicron COVID-19 among elderly individuals in China. Their findings underscore the continued relevance of VDD as a clinical risk factor despite changing viral characteristics and public health policies. Whether the previously identified risk factors, including VDD, maintain clinical significance in this new phase remains uncertain. To address this knowledge gap, this study aimed to investigate the association between VDD and clinical outcomes in COVID-19 patients during the post-Omicron phase (June 2022–December 2023), comparing these findings with data from the pre-Omicron period.

## 2 Methods

### 2.1 Data sources

This retrospective study utilized data from the TriNetX Analytics Network, which provides only de-identified patient counts and statistical summaries without direct interactions with individuals or interventions (23). TriNetX hosts de-identified data from over 157 million unique patients, encompassing demographic details, diagnoses [coded using the International Classification of Diseases (ICD)], procedures [classified by the Current Procedural Terminology (CPT)], medications, laboratory results, and clinical observations. The platform facilitates real-time access to patient data while upholding stringent privacy safeguards. It also offers integrated analytical tools for cohort selection, comparative analyses, and outcome assessments. Although individual patient-level data remain inaccessible, researchers can examine aggregated populations and their associated clinical outcomes. This study was conducted in compliance with the ethical principles of the Declaration of Helsinki and was approved by the Institutional Review Board of the Chi Mei Medical Center (Approval No. 11310-E04), which waived the requirement for informed consent.

### 2.2 Study design

To investigate the association between VDD and clinical outcomes in COVID-19 patients during the Post-Omicron Phase, we conducted a retrospective cohort study. The primary cohort included adult patients treated between June 2022 and December 2023. Patients were stratified into two groups based on their vitamin D status: vitamin D-deficient (VDD group) and normal vitamin D levels (control group). We compared the 30-day clinical outcomes between the VDD and control groups. For historical comparison, we analyzed a second cohort of adult patients from January 2020 to December 2021 (pre-Omicron phase), applying identical inclusion and exclusion criteria. This allowed us to evaluate whether the impact of vitamin D status on clinical outcomes differed between the epidemic phases.

### 2.3 Inclusion criteria

Patients were eligible for inclusion if they were aged 18 years or older at the time of COVID-19 diagnosis and had documented vitamin D levels within one month before infection. VDD was defined as a serum 25-hydroxyvitamin D (25(OH)D) concentration of less than 20 ng/ml, and these patients were categorized into the VDD group. In contrast, patients with a serum 25(OH)D concentration greater than 30 ng/ml were classified as the control group, representing individuals with sufficient vitamin D levels. This classification was implemented to facilitate a comparative analysis of the clinical outcomes between individuals with deficient and adequate vitamin D status. Patients with intermediate vitamin D levels (20–30 ng/mL) were excluded to ensure a clear distinction between the two groups.

### 2.4 Outcomes

The primary outcome was all-cause mortality within 30 days after COVID-19 diagnosis (defined by ICD-10 code for deceased status), while secondary outcomes included pneumonia (ICD-10: J18), respiratory failure (ICD-10: J96.0 or J96), intensive care unit (ICU) admission (identified by CPT code 1013729), acute kidney injury (AKI) (ICD-10: N17), and sepsis (ICD-10: A41.9). All diagnoses were extracted from electronic health records using ICD-10 or CPT codes.

### 2.5 Propensity match score

To minimize potential confounding factors, we performed propensity score matching between the VDD and control groups using a 1:1 nearest-neighbor matching algorithm with a caliper width of 0.1. The matching variables included demographics (age, sex, and race), comorbidities [hypertension (I10), malignancy (C00–D49), obesity (E66), diabetes (E08–E13), smoking status (F17.210), coronary artery disease (I20–I25), chronic kidney disease (N18), alcohol use disorder (F10), cerebrovascular disease (I60–I69), COPD (J44), malnutrition (E40–E46), and liver disease (K76)], COVID-19 vaccination status (J07BN), and baseline laboratory values (BMI, albumin, hemoglobin, eGFR, and HbA1c). Balance between matched groups was assessed using standardized mean differences, with values < 0.1 considered indicative of good balance. The same matching procedure was applied to the cohorts in both the post-Omicron and pre-Omicron phases.

### 2.6 Statistical analysis

All statistical analyses were conducted using the built-in tools of the TriNetX Analytics Platform, as previously described (24–26). Continuous variables are expressed as mean ± standard deviation, and categorical variables are presented as frequencies with percentages. The association between VDD and clinical outcomes was evaluated using logistic regression analysis, which provided odds ratios (ORs) with 95% confidence intervals (CIs). The quality of propensity score matching was assessed through standardized mean differences (SMD) calculated by the platform, with values less than 0.1 indicating adequate balance between matched cohorts. For the analysis of mortality risk factors among patients with VDD, we used TriNetX's multivariate logistic regression module. The model incorporated age, sex, vaccination status, and comorbidities. The results were expressed as adjusted odds ratios (aORs) with corresponding 95% CIs. Statistical significance was set at *p* < 0.05 for the primary outcome and *p* < 0.01 for secondary outcomes to account for multiple comparisons (Bonferroni correction for five secondary outcomes).

## 3 Results

### 3.1 Patient selection and baseline characteristics in the post-Omicron phase

**Figure 1:**
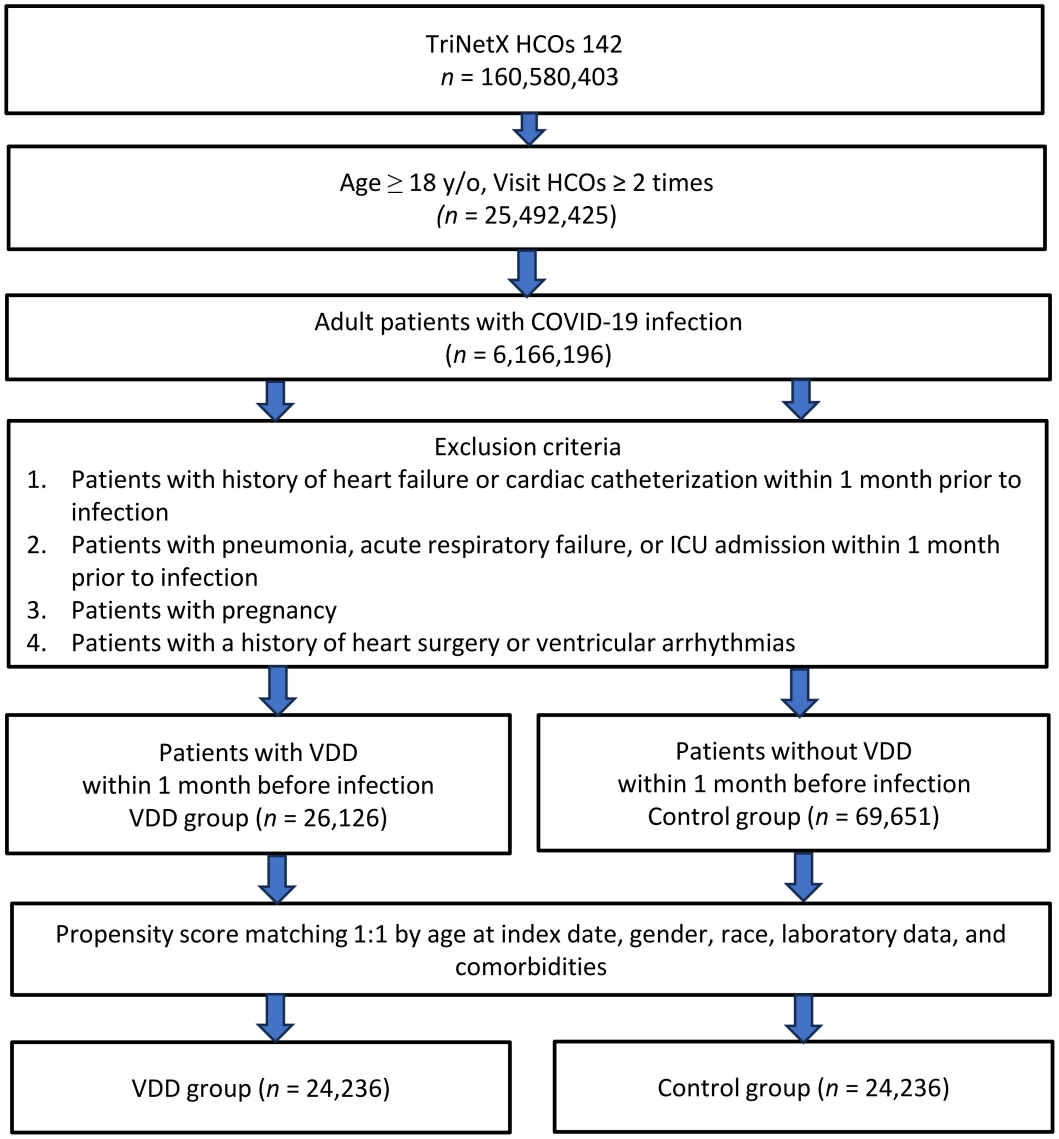
Study flowchart showing the selection process of patients from the TriNetX healthcare database. HCOs, healthcare organizations; ICU, intensive care unit; VDD, vitamin D deficiency.

Among the 160,580,403 patients from 142 TriNetX healthcare organizations, 25,492,425 adult patients had ≥2 healthcare visits (Figure 1). Of these, 6,166,196 were diagnosed with COVID-19 in the post-Omicron phase. After applying the exclusion criteria, we identified 26,126 patients with VDD (VDD group) and 69,651 patients without VDD (control group). Propensity score matching yielded 24,236 matched pairs in the final analysis. After matching, the mean age of patients was 51.3 years in both groups, with approximately 64% being female (Table 1). The racial distribution showed that approximately 52% of the patients were white in both groups. Regarding comorbidities, hypertension was the most prevalent condition, followed by overweight/obesity and neoplasms. Other notable comorbidities included diabetes mellitus, chronic kidney disease, and liver disease. Laboratory data revealed that about 81% of patients had hemoglobin levels > 12 mg/dl, and 83% had albumin levels >3.5g/dl. The mean eGFR was approximately 82.9 and 80.7 ml/min/1.73m^2^ in the VDD and control groups, respectively. COVID-19 vaccination rates were similar between the groups (11.7 vs. 11.2%). All baseline characteristics were well balanced after propensity score matching, with SMDs consistently below 0.1 across all variables (range: 0.001–0.096), indicating excellent covariate balance between groups. Similarly, the baseline characteristics of the patients in the pre-Omicron phase were well balanced across key demographic and clinical variables, ensuring comparability between the groups (Table 2).

**Table 1:** Characteristics of patients before and after matching in the post-Omicron phase.

**Variables**	**Before matching**			**After matching**		
	**VDD group^†^** **(*****n*** = **26,126)**	**Control group (*****n*** = **69,651)**	**SMD**	**VDD group^†^** **(*****n*** = **24,236)**	**Control group (*****n*** = **24,236)**	**SMD**
**Patient characteristics**						
Age at index (years)	50.1 ± 18.6	59.0 ± 17.4	0.491	51.3 ± 18.4	51.3 ± 18.0	<0.001
Female	16,291 (62.4%)	49,474 (71.0%)	0.185	15,435 (63.7%)	15,382 (63.5%)	0.005
White	12,789 (49.0%)	51,188 (73.5%)	0.520	12,650 (52.2%)	12,673 (52.3%)	0.002
Body mass index > 30 kg/m^2^	10,281 (39.4%)	23,430 (33.6%)	0.119	9,385 (38.7%)	9,428 (38.9%)	0.004
Factors influencing health status and contact with health services	20,251 (77.5%)	54,980 (78.9%)	0.034	18,783 (77.5%)	18,802 (77.6%)	0.002
**Comorbidities**						
Essential (primary) hypertension	9,744 (37.3%)	31,224 (44.8%)	0.154	9,234 (38.1%)	9,339 (38.5%)	0.009
Overweight and obesity	7,064 (27.0%)	15,216 (21.8%)	0.121	6,406 (26.4%)	6,486 (26.8%)	0.007
Neoplasms	6,535 (25.0%)	23,289 (33.4%)	0.186	6,311 (26.0%)	6,203 (25.6%)	0.010
Diabetes mellitus	5,374 (20.6%)	13,702 (19.7%)	0.022	4,971 (20.5%)	4,963 (20.5%)	0.001
Chronic kidney disease (CKD)	3,145 (12.0%)	9,594 (13.8%)	0.052	2,934 (12.1%)	2,874 (11.9%)	0.008
Liver diseases	2,434 (9.3%)	5,508 (7.9%)	0.050	2,179 (9.0%)	2,090 (8.6%)	0.013
Ischemic heart diseases	2,172 (8.3%)	7,428 (10.7%)	0.080	2,075 (8.6%)	1,994 (8.2%)	0.012
Nicotine dependence	2,180 (8.3%)	3,332 (4.8%)	0.144	1,814 (7.5%)	1,764 (7.3%)	0.008
Cerebrovascular diseases	1,820 (7.0%)	5,010 (7.2%)	0.009	1,672 (6.9%)	1,607 (6.6%)	0.011
Malnutrition	1,450 (5.6%)	2,439 (3.5%)	0.099	1,205 (5.0%)	1,141 (4.7%)	0.012
COPD	1,125 (4.3%)	3,506 (5.0%)	0.034	1,070 (4.4%)	1,068 (4.4%)	<0.001
Alcohol related disorders	1,383 (5.3%)	1,738 (2.5%)	0.145	1,061 (4.4%)	1,053 (4.3%)	0.002
COVID-19 vaccines	2,946 (11.3%)	11,057 (15.9%)	0.135	2,846 (11.7%)	2,723 (11.2%)	0.016
**Laboratory data**						
Hemoglobin >12mg/dl	20,656 (79.1%)	59,749 (85.8%)	0.177	19,533 (80.6%)	19,621 (81.0%)	0.009
Albumin >3.5mg/dl	21,296 (81.5%)	62,089 (89.1%)	0.217	20,168 (83.2%)	20,151 (83.1%)	0.002
Hemoglobin A1c >7%	3,036 (11.6%)	7,085 (10.2%)	0.046	2,787 (11.5%)	2,784 (11.5%)	<0.001
eGFR ml/min/1.73 m^2^	84.0 ± 36.0	75.9 ± 26.4	0.257	82.9 ± 35.0	80.7 ± 29.2	0.067

† Patients who had a confirmed diagnosis of COVID-19 within 30 days postoperatively.

SMD, standardized mean differences; COPD, chronic obstructive pulmonary disease; eGFR, estimated glomerular filtration rate.

**Table 2:** Characteristics of patients before and after matching in the pre-Omicron phase.

**Variables**	**Before matching**			**After matching**		
	**VDD group^†^** **(*****n*** = **26,126)**	**Control group (*****n*** = **69,651)**	**SMD**	**VDD group^†^** **(*****n*** = **24,236)**	**Control group (*****n*** = **24,236)**	**SMD**
**Patient characteristics**						
Age at index (years)	50.9 ± 19.2	59.0 ± 17.3	0.445	52.7± 18.6	52.2 ± 18.7	0.027
Female	17,734 (61.8%)	51,406 (71.1%)	0.198	14,579 (64.4%)	14,529 (64.2%)	0.005
White	13,970 (48.6%)	53,203 (73.5%)	0.528	12,251 (54.1%)	12,353 (54.6%)	0.009
Body mass index > 30 kg/m^2^	9,463 (33.0%)	24,603 (34.0%)	0.022	8,209 (36.3%)	8,378 (37.0%)	0.015
Factors influencing health status and contact with health services	18,018 (62.7%)	57,643 (79.7%)	0.381	15,917 (70.3%)	16,102 (71.1%)	0.018
**Comorbidities**						
Essential (primary) hypertension	8,269 (28.8%)	32,675 (45.2%)	0.344	7,699 (34.0%)	7,747 (34.2%)	0.004
Overweight and obesity	5,684 (19.8%)	16,030 (22.2%)	0.058	5,046 (22.3%)	5,067 (22.4%)	0.002
Neoplasms	5,526 (19.2%)	23,988 (33.2%)	0.321	5,165 (22.8%)	5,204 (23.0%)	0.004
Diabetes mellitus	4,568 (15.9%)	14,327 (19.8%)	0.102	4,159 (18.4%)	4,079 (18.0%)	0.009
Chronic kidney disease (CKD)	2,618 (9.1%)	9,996 (13.8%)	0.148	2,374 (10.5%)	2,275 (10.0%)	0.014
Liver diseases	1,762 (6.1%)	5,817 (8.0%)	0.074	1,617 (7.1%)	1,566 (6.9%)	0.009
Ischemic heart diseases	1,659 (5.8%)	7,904 (10.9%)	0.187	1,581 (7.0%)	1,584 (7.0%)	0.001
Nicotine dependence	1,646 (5.7%)	3,410 (4.7%)	0.046	1,416 (6.3%)	1,351 (6.0%)	0.012
Cerebrovascular diseases	1,328 (4.6%)	5,252 (7.3%)	0.112	1,227 (5.4%)	1,218 (5.4%)	0.002
Malnutrition	772 (2.7%)	2,567 (3.5%)	0.050	699 (3.1%)	713 (3.2%)	0.004
COPD	928 (3.2%)	3,644 (5.0%)	0.091	869 (3.8%)	834 (3.7%)	0.008
Alcohol related disorders	998 (3.5%)	1,810 (2.5%)	0.057	797 (3.5%)	735 (3.2%)	0.015
COVID-19 vaccines	1,894 (6.6%)	12,410 (17.2%)	0.331	1,787 (7.9%)	1,717 (7.6%)	0.012
**Laboratory data**						
Hemoglobin >12mg/dL	18,460 (64.3%)	62,168 (85.9%)	0.517	17,326 (76.5%)	17,403 (76.9%)	0.008
Albumin >3.5mg/dL	18,086 (63.0%)	64,687 (89.4%)	0.653	17,398 (76.9%)	17,495 (77.3%)	0.010
Hemoglobin A1c >7%	2,599 (9.1%)	7,332 (10.1%)	0.037	2,393 (10.6%)	2,369 (10.5%)	0.003
eGFR mL/min/1.73 m^2^	81.1 ± 33.8	75.9 ± 26.2	0.169	80.8 ± 33.2	80.2 ± 8.7	0.019

† Patients who had a confirmed diagnosis of COVID-19 within 30 days postoperatively.

SMD, standardized mean differences; COPD, chronic obstructive pulmonary disease; eGFR, estimated glomerular filtration rate.

### 3.2 Primary and secondary outcomes

In the post-Omicron phase (2022–2024), patients with VDD demonstrated significantly higher rates of adverse events than the control group (Table 3). The primary outcome of mortality was notably higher in the VDD group (0.89% vs. 0.49%, OR 1.82, 95% CI 1.46–2.28, *p* < 0.001). Among the secondary outcomes, AKI showed the highest incidence in both groups, with a substantially higher rate in patients with VDD (5.08% vs. 3.66%, OR 1.41, 95% CI 1.29–1.54, *p* < 0.001). Respiratory complications were also more frequent in the VDD group, including respiratory failure (1.81% vs. 1.36%, OR 1.34, 95% CI 1.16–1.54, *p* < 0.001), and pneumonia (1.80% vs. 1.47%, OR 1.23, 95% CI 1.07–1.42, *p* = 0.004). Similarly, ICU admission (1.73% vs. 1.33%, OR 1.04, 95% CI 1.13–1.51, *p* < 0.001) and sepsis (1.80% vs. 1.49%, OR 1.21, 95% CI 1.06–1.40, *p* = 0.007) occurred more frequently in patients with VDD. These findings consistently demonstrated that VDD was associated with worse clinical outcomes across all measured parameters.

**Table 3:** Primary and secondary outcomes in patients with or without vitamin D deficiency at 30-day follow-up in the post-Omicron phase.

**Outcomes**	**VDD group (*n* = 24,236)**	**Control group (*n* = 24,236)**	**OR (95% CI)^‡^**	***p*-value**
	**Events (%)**	**Events (%)**		
Mortality	216 (0.89%)	119 (0.49%)	1.82 (1.46, 2.28)	<0.001
Pneumonia	437 (1.80%)	356 (1.47%)	1.23 (1.07, 1.42)	0.004
Respiratory failure	439 (1.81%)	330 (1.36%)	1.34 (1.16, 1.54)	<0.001
ICU admission	420 (1.73%)	323 (1.33%)	1.04 (1.13, 1.51)	<0.001
AKI	1,231 (5.08%)	887 (3.66%)	1.41 (1.29, 1.54)	<0.001
Sepsis	437 (1.80%)	361 (1.49%)	1.21 (1.06, 1.40)	0.007

AKI, acute kidney injury; VDD, vitamin D deficiency; ICU, intensive care unit.

‡ Control group as reference.

In the pre-Omicron phase (2020–2021), VDD was associated with markedly higher adverse outcomes compared to controls in a matched cohort of 22,638 pairs (Table 4). The mortality rate was significantly higher in the VDD group (1.43% vs. 0.39%; OR, 3.67; 95% CI 2.90–4.64, *p* < 0.001). Secondary outcomes showed similar patterns: AKI (6.23% vs. 3.06%, OR 2.11), respiratory failure (2.20% vs. 1.33%, OR 1.66), ICU admission (1.86% vs. 1.09%, OR 1.71), pneumonia (1.99% vs. 1.49%, OR 1.34), and sepsis (2.17% vs. 1.37%, OR 1.60), all with *p* < 0.001. Notably, the risks for adverse outcomes were consistently higher in the pre-Omicron phase compared to the post-Omicron phase, particularly for mortality (OR 3.67 vs. 1.82).

**Table 4:** Primary and secondary outcomes at 30-day follow-up in the pre-Omicron phase (2020–2021).

**Outcomes**	**VDD group (*n* = 22,638)**	**Control group (*n* = 22,638)**	**OR (95% CI)^‡^**	***p*-value**
	**Events (%)**	**Events (%)**		
Mortality	323 (1.43%)	89 (0.39%)	3.67 (2.90, 4.64)	<0.001
Pneumonia	450 (1.99%)	337 (1.49%)	1.34 (1.16, 1.55)	<0.001
Respiratory failure	498 (2.20%)	302 (1.33%)	1.66 (1.44, 1.92)	<0.001
ICU admission	420 (1.86%)	247 (1.09%)	1.71(1.46, 2.01)	<0.001
AKI	1,410 (6.23%)	692 (3.06%)	2.11 (1.92, 2.31)	<0.001
Sepsis	491 (2.17%)	309 (1.37%)	1.60 (1.39, 1.85)	<0.001

AKI, acute kidney injury; VDD, vitamin D deficiency; ICU, intensive care unit.

‡ Control group as reference.

### 3.3 Risk factors for mortality in patients with vitamin D deficiency

In analyzing the risk factors for mortality among patients with VDD, several consistent patterns emerged across both the pre-Omicron and post-Omicron phases (Table 5). Age remained a significant risk factor in both periods. Female sex and COVID-19 vaccination consistently demonstrated protective effects across both phases, with females having approximately half the risk of mortality (post-Omicron: aOR 0.50; pre-Omicron: aOR 0.41) and vaccination showing similar protective effects (post-Omicron: aOR 0.50; pre-Omicron: aOR 0.52). Among comorbidities, malnutrition emerged as the strongest risk factor, with a notably higher risk in the post-Omicron phase (aOR 4.34, 95% CI 3.18–5.92) compared to the pre-Omicron phase (aOR 2.83, 95% CI 1.90–4.22). Neoplasms and cerebrovascular diseases also showed consistently elevated risks across both phases, though with higher odds ratios in the post-Omicron period (neoplasms: aOR 2.63 vs. 1.62; cerebrovascular diseases: aOR 2.16 vs. 1.54). Liver diseases showed a more pronounced association with mortality in the post-Omicron phase (aOR 3.08, 95% CI 2.23–4.25) compared to the pre-Omicron period (aOR 1.49, 95% CI 1.00–2.22). Interestingly, obesity demonstrated a protective effect in both phases (post-Omicron: aOR, 0.36; pre-Omicron: aOR, 0.26). Several comorbidities, including diabetes mellitus, nicotine dependence, and alcohol-related disorders, were not significantly associated with mortality in either phase.

**Table 5:** Risk factors for mortality in patients with vitamin D deficiency in the pre-Omicron and post-Omicron phases.

**Variables**	**Post-Omicron phase (2022–2024)**		**Pre-Omicron phase (2020–2021)**	
	**aOR (95% CI)**	***p*** **-value**	**aOR (95% CI)**	***p*** **-value**
Age at index	1.05 (1.04, 1.06)	<0.001	1.07 (1.06, 1.07)	<0.001
Female	0.50 (0.38, 0.65)	<0.001	0.41 (0.33, 0.50)	<0.001
White	1.20 (0.92, 1.56)	0.184	1.09 (0.89, 1.33)	0.413
Essential (primary) hypertension	0.73 (0.54, 0.98)	0.037	0.83 (0.64, 1.06)	0.14
Neoplasms	2.63 (2.01, 3.45)	<0.001	1.62 (1.30, 2.02)	<0.001
Overweight and obesity	0.36 (0.23, 0.56)	<0.001	0.26 (0.15, 0.44)	<0.001
Diabetes mellitus	0.95 (0.69, 1.32)	0.768	0.93 (0.69, 1.25)	0.632
Nicotine dependence	0.76 (0.50, 1.16)	0.198	0.77 (0.49, 1.19)	0.233
Ischemic heart diseases	0.91 (0.63, 1.31)	0.599	1.04 (0.74, 1.45)	0.840
Chronic kidney disease (CKD)	1.28 (0.92, 1.78)	0.151	0.94 (0.68, 1.31)	0.728
Alcohol related disorders	0.98 (0.62, 1.55)	0.929	1.11 (0.67, 1.19)	0.685
Cerebrovascular diseases	2.16 (1.55, 3.01)	<0.001	1.54 (1.10, 2.16)	0.011
Chronic obstructive pulmonary disease	1.58 (1.03, 2.45)	0.038	1.26 (0.82, 1.95)	0.290
Malnutrition	4.34 (3.18, 5.92)	<0.001	2.83 (1.90, 4.22)	<0.001
Liver diseases	3.08 (2.23, 4.25)	<0.001	1.49 (1.00, 2.22)	0.050
COVID-19 vaccines	0.50 (0.31, 0.82)	0.005	0.52 (0.31, 0.88)	0.015
Anemia	1.07 (0.78, 1.46)	0.695	1.17 (0.85, 1.61)	0.334

aOR, adjusted odds ratio.

## 4 Discussion

Analysis of propensity-matched cohorts from the TriNetX Analytics Network revealed significant associations between VDD and adverse COVID-19 outcomes in both the pre- and post-Omicron phases. The impact of VDD was more pronounced during the pre-Omicron period across all measured outcomes, particularly mortality and AKI. In the post-Omicron phase, although the association remained significant, the magnitude of the effect decreased substantially. Risk factor analysis identified malnutrition as the strongest predictor of mortality among patients with VDD, and its effect intensified in the post-Omicron phase. Female sex and COVID-19 vaccination demonstrated consistent protective effects in both the periods. Notably, obesity has emerged as a protective factor, contrary to traditional risk patterns. The transition from the pre- to post-Omicron phases was marked by evolving risk patterns, particularly in the increased significance of liver disease and the sustained importance of pre-existing conditions, such as neoplasms and cerebrovascular diseases.

The relationship between VDD and COVID-19 complications has been extensively studied during the early phases of the pandemic, with multiple studies suggesting an increased risk of severe disease and mortality among vitamin D-deficient patients (27, 28). However, these findings primarily reflect the pre-Omicron viral strains, which demonstrated different pathogenicity and clinical manifestations compared to the current variants. The emergence of Omicron and its subvariants, combined with widespread vaccination and evolving treatment protocols, has fundamentally altered the COVID-19 pandemic. This shift raises important questions regarding whether the previously identified risk factors maintain their clinical significance. Given that vitamin D measurement requires additional healthcare resources and is not routinely performed in all COVID-19 patients, establishing its continued relevance in the post-Omicron phase is crucial for evidence-based clinical decision-making.

The relationship between VDD and COVID-19 mortality demonstrated significant evolution across pandemic phases, revealing both consistency with previous research and novel insights (28, 29). Our finding of increased mortality risk among patients with VDD in the pre-Omicron phase aligns with those of earlier studies (28, 29). However, our study extends these previous findings by demonstrating that while VDD remains a significant risk factor in the post-Omicron phase, its impact is notably attenuated. This temporal change in the strength of the association represents a novel finding with important implications. The reduced magnitude of the effect of vitamin D in the post-Omicron phase likely reflects the interplay of multiple factors, including the emergence of less virulent viral strains, improved treatment protocols, and widespread vaccination coverage. Our observation that the protective effect of vaccination remains consistent across both phases in patients with VDD is particularly noteworthy, as it suggests independent but complementary protective mechanisms.

Our analysis revealed an important pattern in the interaction between vitamin D status and other risk factors. The heightened impact of malnutrition and liver disease in patients with VDD during the post-Omicron phase is an unreported finding. This observation suggests that as the virus has evolved, the influence of metabolic and nutritional factors on disease outcomes may have become more prominent. The persistent protective effect of the female sex across both phases adds to our understanding of sex-based differences in COVID-19 outcomes and their interaction with nutritional status. Our findings have several important clinical implications. First, they supported continuing vitamin D screening in high-risk COVID-19 patients, even in the context of less severe variants. The strong association between VDD and mortality in patients with specific comorbidities (particularly malnutrition and liver disease) suggests the need for targeted screening. Second, the attenuated but persistent mortality risk highlights the potential value of vitamin D supplementation strategies (30, 31), particularly in vulnerable populations. Third, the consistent protective effect of vaccination in patients with VDD emphasizes the importance of maintaining preventive measures, regardless of nutritional status. The evolution of observed risk patterns calls for a dynamic approach to patient risk stratification. Although VDD may no longer carry the same magnitude of risk as in the earlier pandemic phases, its continued association with mortality suggests the need for ongoing vigilance. Healthcare providers should consider vitamin D status as part of a comprehensive risk assessment, particularly in patients with malnutrition, liver disease, or other identified risk factors. Future research should focus on determining the optimal vitamin D screening strategies in the post-Omicron era and investigating whether targeted supplementation in high-risk groups could modify outcomes.

The association between VDD and increased pneumonia risk demonstrates interesting temporal changes across the pandemic phases. Although the risk of pneumonia remained elevated in patients with VDD during both periods, the attenuated association in the post-Omicron phase aligns with the generally milder respiratory manifestations reported for the Omicron variants. The persistence of elevated pneumonia risk, albeit reduced, suggests that the role of vitamin D in maintaining respiratory epithelial barrier function and modulating inflammatory responses remains relevant, even with evolved viral strains. This has important clinical implications, particularly for patients with pre-existing respiratory diseases or other risk factors for severe diseases. These findings support maintaining vigilance for respiratory complications in patients with VDD, even in the context of presumably milder Omicron variants, while also suggesting that preventive strategies may require recalibration based on individual risk profiles.

Our findings have important implications for public health policy and clinical practice, particularly in low-resource settings where healthcare access and diagnostic capacity may be limited. Given the consistent association between vitamin D deficiency and adverse COVID-19 outcomes, even during the post Omicron phase, our results support the integration of targeted vitamin D screening into clinical risk assessment protocols for high-risk populations. These groups may include individuals with malnutrition, liver disease, or chronic illnesses or older adults who are more vulnerable to poor outcomes. In settings where universal screening is not feasible due to economic or logistical constraints, a focused, risk-based screening strategy may provide a cost-effective and pragmatic approach to identify individuals most likely to benefit from early intervention. Moreover, incorporating low-cost vitamin D supplementation into routine care or community-based public health programs could offer a scalable and preventive measure to reduce disease burden. This approach may be especially valuable in regions where nutritional deficiencies are common and the healthcare infrastructure is under strain. Overall, our findings underscore the potential role of vitamin D status as a modifiable factor in COVID-19 management, and advocate tailored screening and supplementation strategies within diverse healthcare systems.

Several important limitations of this study should be considered when interpreting these findings. First, the retrospective nature of this study inherently introduces a potential selection bias, as vitamin D levels were only available for patients who had undergone testing, possibly overrepresenting individuals with specific health concerns or greater access to healthcare. Furthermore, our reliance on electronic health records, while providing a large sample size, may not capture all relevant clinical details or confounding factors that could influence the outcomes. Second, the timing of vitamin D measurement presents another consideration, as levels were assessed within one month before COVID-19 diagnosis. This single time-point measurement may not fully reflect the dynamic nature of vitamin D status or account for seasonal variation. Additionally, we could not account for unreported vitamin D supplementation or dietary intake that might have occurred after testing but before or during COVID-19 infection. Third, the evolving nature of COVID-19 treatment protocols during the study period may have influenced the outcomes independently of vitamin D status. Although we attempted to control for vaccination status, the varying effectiveness of different vaccine types and the timing of vaccination relative to infection could not be fully addressed in our analysis. Furthermore, the emergence of different Omicron subvariants during the study period may have introduced additional variability in disease severity and outcomes. Finally, the generalizability of our findings may be limited by the characteristics of the TriNetX network population, which may not fully represent all demographic groups or healthcare settings. To address the limitations of our retrospective study, future research should include prospective cohort studies or randomized controlled trials to better establish temporal relationships and causality. Such designs would allow for more rigorous control of confounding variables, standardized data collection, and comprehensive follow-up. Interventional studies, particularly those evaluating the effects of vitamin D supplementation in high-risk populations, could provide clearer evidence regarding the potential benefits of modifying vitamin D status on COVID-19 outcomes.

## 5 Conclusion

This study demonstrates that VDD continues to influence COVID-19 outcomes in the post-Omicron era, albeit with a reduced impact compared to earlier pandemic phases. The evolving relationship between vitamin D status and disease severity, coupled with shifting patterns of risk factors, suggests the need for dynamic approaches to patient risk assessment and management. Although the nature of the pandemic has changed, attention to vitamin D status remains clinically relevant. These findings support the integration of vitamin D screening into COVID-19 management protocols, while emphasizing the need for comprehensive risk assessment that considers both traditional and emerging factors. Future prospective studies should investigate the potential benefits of vitamin D supplementation in high-risk populations during the evolving phase of the pandemic.

## Data Availability

The raw data supporting the conclusions of this article will be made available by the authors, without undue reservation.
